# Alterations in gastric and gut microbiota following sleeve gastrectomy in high-fat diet-induced obese rats

**DOI:** 10.1038/s41598-023-48718-w

**Published:** 2023-12-02

**Authors:** Young Suk Park, Kung Ahn, Kyeongeui Yun, Jinuk Jeong, Kyung-Wan Baek, Jieun Lee, Hyung-Ho Kim, Kyudong Han, Yong Ju Ahn

**Affiliations:** 1 Department of Surgery, Seoul National University Bundang Hospital, Seongnam, Gyeonggi-do South Korea; 2 Department of Surgery, Seoul National University College of Medicine, Seoul, South Korea; 3 HuNbiome Co., Ltd, R&D Center, Gasan Digital 1-ro, Geumcheon-gu, Seoul, South Korea; 4 Department of Bioconvergence Engineering, Dankook University, Yongin, 1491 South Korea; 5 Research Institute of Pharmaceutical Sciences, Gyeongsang National University, Jinju, South Korea; 6 Center for Bio-Medical Engineering Core Facility, Dankook University, Cheonan, 31116 South Korea; 7 Department of Microbiology, College of Science and Technology, Dankook University, Cheonan, 31116 South Korea

**Keywords:** Microbiology, Clinical microbiology, Microbial communities

## Abstract

Obesity is considered a high-risk disease and a global epidemic, and the number of obese patients is rising at an alarming rate worldwide. High-fat diet-induced dysbiosis of the intestinal microbiota is considered an essential factor related to obesity. Bariatric surgery induces a sharp decrease in fat content and effectively improves the metabolism of obese individuals. Herein, we aimed to investigate the effects of a high-fat diet-induced obesity and the alterations in gastric and intestinal microbiota resulting from sleeve gastrectomy on clinical outcomes. We performed 16S sequencing of gastric and fecal samples obtained from rats in three treatment groups: normal chow diet, high-fat diet (HFD), and sleeve gastrectomy after HDF for 14 weeks. The area under the curve of fasting glucose and the levels of leptin and low-density lipoproteins were significantly different between groups. Microbial taxa that were highly correlated with several clinical parameters were identified for each group. Glyoxylate and dicarboxylate, taurine and hypotaurine, butanoate, nitrogen, and pyrimidine metabolism and aminoacyl-transfer ribonucleic acid biosynthesis were affected by bariatric surgery and were significantly associated with changes in the composition of gastric and fecal microbiomes. Connectivity and co-occurrence were higher in fecal samples than in gastric tissues. Our results elucidated the positive effects of sleeve gastrectomy in obesity and shed light on changes in the microbiomes of gastric and fecal samples.

## Introduction

In recent years, most of the world with the exception of some developing countries has seen rapid industrialization and urbanization, accompanied by an increase in the personal income and living standards of people^1–3^. This social transformation has led to changes in the dietary habits of people by affecting economic growth in the region they inhabit^4^. Instant foods (such as fast food) have gained popularity, and high-protein and high-fat diets have become entrenched in some populations^5,6^. A high-fat diet is closely related to the induction of obesity, which causes various diseases in adults^7,8^. Obesity has been treated as a high-risk disease in public health and as a global epidemic in the last few decades; at present, the number of obese patients continues to rise at an alarming rate worldwide^9^. Obesity not only exhibits the intuitive morphology of overweight but also is closely related to pathological conditions such as hyperlipidemia and the resulting arteriosclerosis (caused by increased blood cholesterol levels) and type-2 diabetes (caused by insulin resistance)^10,11^. Recent studies have shown that obesity induces inflammation in the adipose tissue, which is also associated with certain cancers^12,13^. Recently, high-fat diet-induced dysbiosis of the intestinal microbiota has been considered an essential factor related to various causes of obesity^14,15^.

Studies by large-scale microbiome consortiums (such as the Human Microbiome Project) have revealed that the intestinal microbial community of humans forms a very complex ecological network consisting of 1014 bacterial cells (400–500 species per gram of colonic content)^16,17^. Recent microbiome studies have reported that the bacterial community of the human intestinal microbiome is mainly composed of species belonging to two phyla: *Bacteroidetes* and *Firmicutes*. A change in the *Bacteroidetes*/*Firmicutes* ratio in the intestine is associated with obesity^18,19^ and is closely related to eating habits. Several researchers in the field of human health care are developing techniques to prevent obesity by balancing the intestinal microbiota and increasing the abundance of beneficial bacteria through dietary correction or probiotic therapy^20–22^. However, these non-clinical methods of preventing obesity have different preventive effects depending on the individual’s lifestyle, dietary habits, and constitution. Therefore, people with severe obesity are treated by controlling fat accumulation in the body through bariatric procedures such as gastric bypass or sleeve gastrectomy (SG).

Bariatric surgery induces a sharp decrease in fat content, effectively improves metabolism^23^, improves sensitivity to lipolysis (controlled by insulin and catecholamine), induces changes in adipokine secretion, and reduces intestinal inflammation by controlling the balance of the immune system^24,25^. Interestingly, researchers have recently hypothesized that bariatric surgery induces changes in the intestinal microbial community by regulating their metabolism^26^. Additionally, changes in the proportions of *Firmicutes* (associated with changes in body weight before and after bariatric surgery) and *Proteobacteria* (associated with the regulation of the inflammatory response and glucose homeostasis) in the intestine are correlated with a decrease in mean body mass index^27^. To date, studies have analyzed the 16S sequences of the fecal microbiome to demonstrate changes in the gut microbiome following dietary changes or bariatric surgery. However, few studies have investigated the association between changes in the fecal and gastric tissue microbiomes before and after bariatric surgery. Sleeve gastrectomy is a type of bariatric surgery that is used to treat obesity by reducing the size of the stomach. Some studies have suggested that this procedure may alter the microbiome, or the community of bacteria that live in the gut^28^.

In this study, we performed a parallel comparison of changes in the microbial composition of the gastric and fecal microbiomes after SG in rats with high-fat diet-induced obesity. We performed 16S metagenome analysis and multiple clinical tests in 25 rats in three groups: high-fat diet (HFD), normal chow diet (NCD), and high-fat diet with SG (sHFD).

## Results

### Differences in clinical parameters between groups

**Figure 1:**
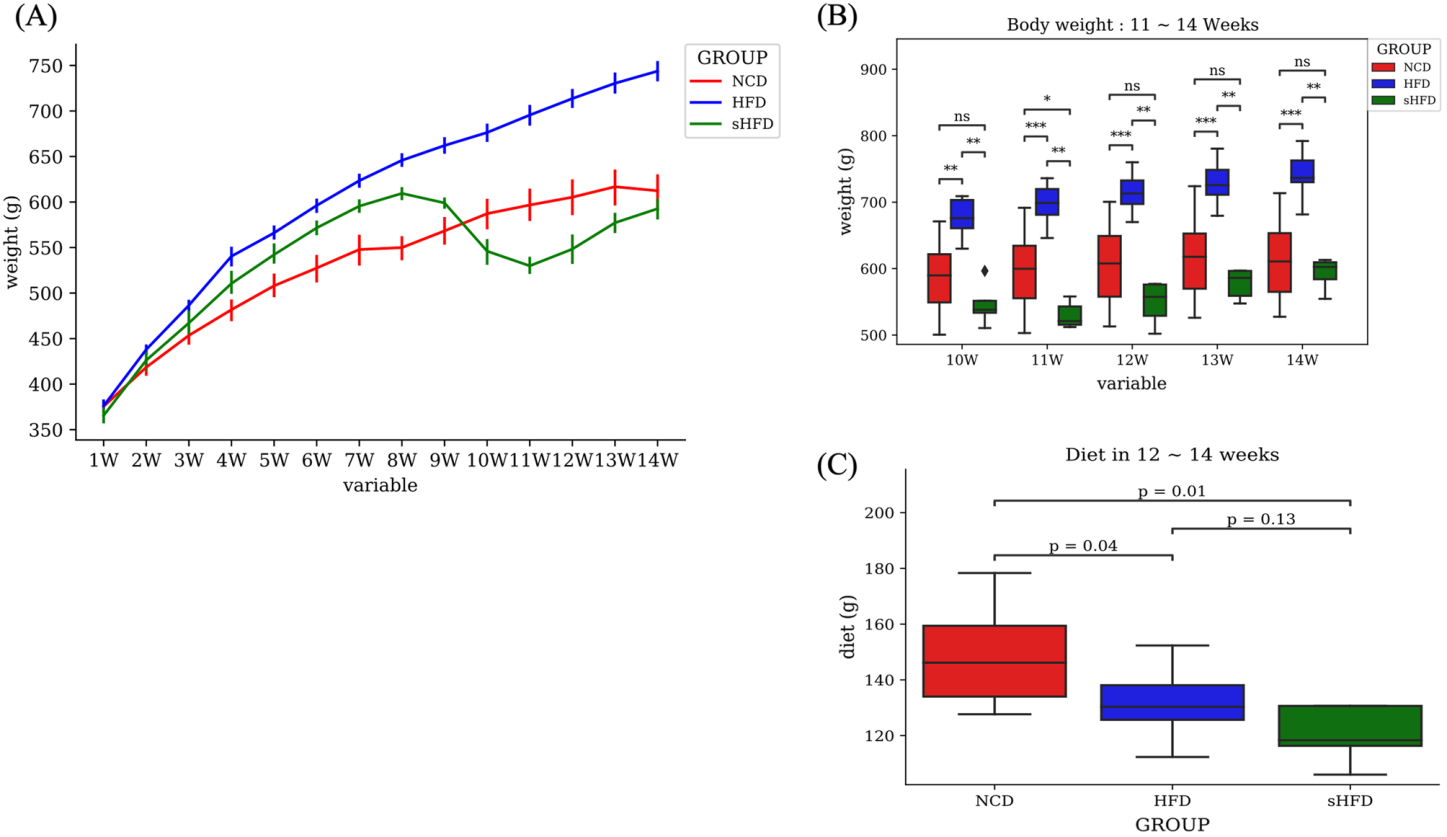
Changes in the body weight and diet intake of rats in the NCD, HFD, and sHFD groups. (**A**) Body weight in each group over a duration of 14 weeks. The blue solid line indicates the high-fat diet (HFD) group. The green and red solid lines indicate the HDF-induced obesity group that underwent gastrectomy (sHFD) and the group fed a normal chow diet (NCD), respectively. NCD: Normal chow diet group, HFD: High fat diet group, sHFD: after high fat diet, gastrectomy group. (**B**) A bar plot showing the body weight of each group from the time of gastrectomy (weeks 10–14). The asterisk (*) represents the *p*-value of the comparative statistical test for each bacterial genera frequency (* < 0.05; ** < 0.01; *** < 0.001). (**C**) Box plot showing the diet intake (g) during weeks 12–14.

Body weight increased significantly during the 14 weeks of feeding in all groups. Body weight was significantly lower in the sHFD group than in the NCD group during weeks 9–11, but showed similar patterns from week 12. During weeks 12–14, food intake was significantly lower in the sHFD group than in the NCD group (*p* = 0.01) and non-significantly lower in the sHFD group than in the HFD group (Fig. 1A–C).

We analyzed the differences in 12 clinical parameters (Supplementary Figure S2, Supplementary Table S2–S4) between three experimental groups (NCD, HFD, and sHFD) and found significant differences in the area under the curve (AUC) of fasting glucose and the levels of HDL, LDL, and leptin. Glucose AUC was significantly higher in the HFD group than in the NCD (*p* < 0.001) and sHFD (*p* < 0.01) groups. HDL levels were significantly higher in the sHFD group than in the NCD (*p* = 0.05) group and non-significantly higher in the sHFD than in the HFD (*p* = 0.06) group. LDL levels were significantly lower in the NCD than in the HFD (*p* < 0.001) and sHFD (*p* < 0.01) groups. Leptin levels were also significantly lower in the NCD than in the HFD (*p* < 0.001) and sHFD (*p* < 0.01) groups (Table 1). Although there were no significant differences in clinical parameters between the HFD and sHFD groups, the levels of leptin decreased (*p* = 0.67) and those of HDL increased after SG in the sHFD group (*p* = 0.06).

**Table 1:** Differences in clinical parameters between the NCD, HFD, sHFD groups.

parameter (unit)	NCD vs HFD vs sHFD	NCD vs HFD	NCD vs sHFD	HFD vs sHFD
GIP (pg/mL)	0.0359(*)	0.684(ns)	0.005(**)	0.075(.)
GLU_AUC (glucose. mg/dL)	0.000163(***)	1.08e-05(***)	0.594(ns)	0.000666(***)
Glucagon (pg/mL)	0.233(ns)	0.28(ns)	0.371(ns)	0.165(ns)
Insulin (pg/mL)	0.392(ns)	0.165(ns)	0.768(ns)	0.679(ns)
Leptin (pg/mL)	0.000798(***)	0.000325(***)	0.001(***)	0.679(ns)
PYY (pg/mL)	0.94(ns)	0.971(ns)	0.859(ns)	0.768(ns)
ALT (U/L)	0.509(ns)	0.325(ns)	0.759(ns)	0.426(ns)
AST (U/L)	0.49(ns)	0.273(ns)	1(ns)	0.462(ns)
GGT (U/L)	0.297(ns)	0.663(ns)	0.134(ns)	0.311(ns)
TG (mg/dL)	0.522(ns)	0.325(ns)	0.426(ns)	0.953(ns)
HDL (mg/dL)	0.0837(.)	0.762(ns)	0.05(*)	0.055(.)
LDL (mg/dL)	0.000858(***)	0.001(***)	0.004(**)	0.297(ns)

The asterisk (*) represents the *p*-value of the statistical test (* < 0.05; ** < 0.01; *** < 0.001; *p* < 0.1). NCD: Normal choe diet group, HFD: High fat diet group, sHFD: after high fat diet, gastrectomy group.

### Diversity Analysis of gastric (gastric tissues) and fecal (gut) microbes across groups

**Figure 2:**
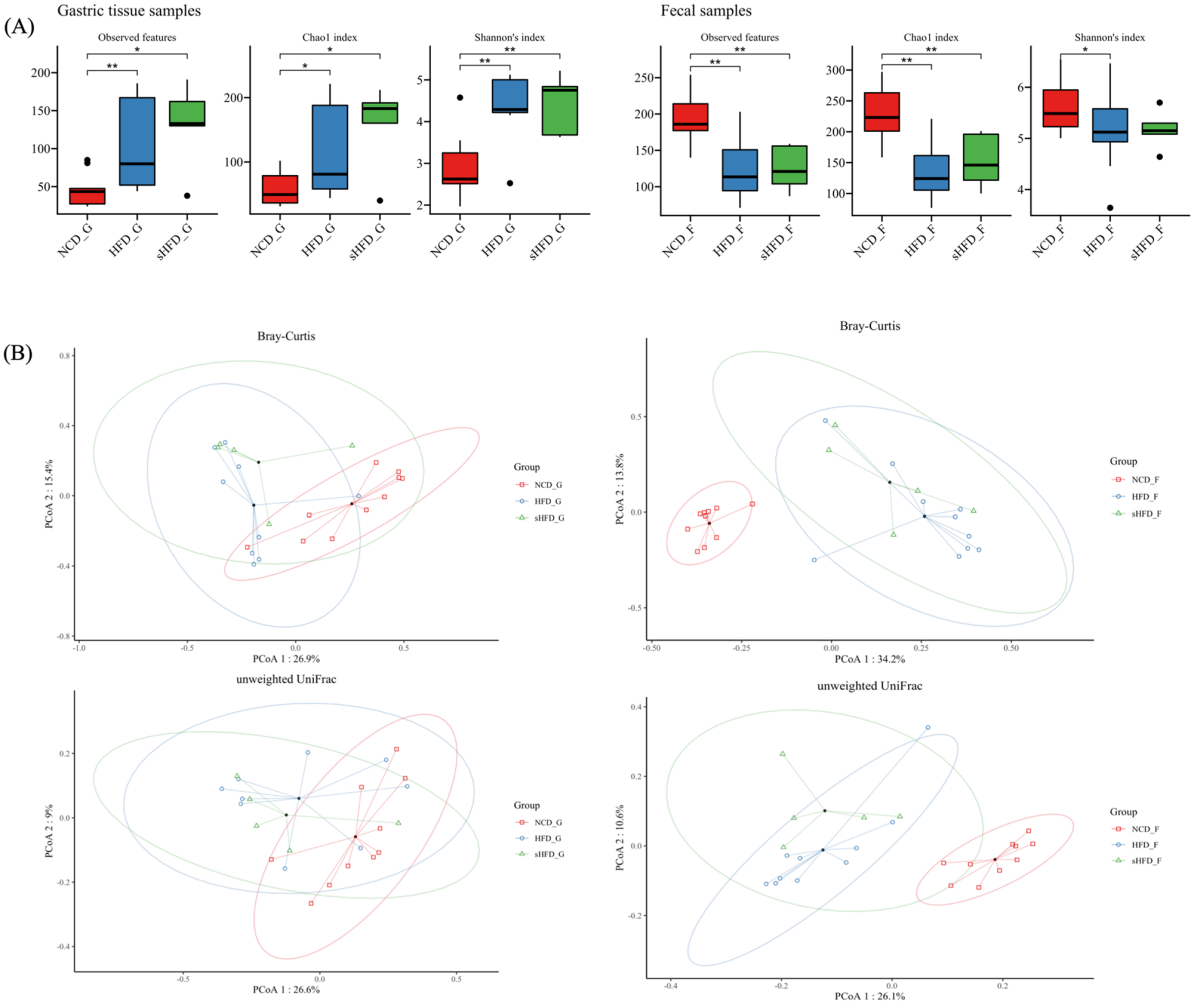
The diversity of gastric- and fecal-derived microbiota in the NCD, HFD, and sHFD groups. (**A**) Alpha diversity indices are shown for the gastric- and fecal-derived microbiota of rats fed the normal chow diet (NCD), rats fed a high-fat diet (HFD), and HFD-induced obese rats that underwent sleeve gastrectomy. (**B**) Principal Coordinate analysis (PCoA) plots showing results of microbial beta-diversity comparisons with the control group (red) for each oral disease group (blue). NCD_G: Gastric derived microbiota in normal chow diet group, HFD_G: Gastric derived microbiota in high-fat diet group, sHFD_G: Gastric derived microbiota in HFD-induced obese rats that underwent sleeve gastrectomy group. NCD_F: Fecal derived microbiota in normal chow diet group, HFD_F: Fecal derived microbiota in high-fat diet group, sHFD_F: Fecal derived microbiota in HFD-induced obese rats that underwent sleeve gastrectomy group.

Community diversity, as estimated by the Chao 1, Shannon, Simpson’s, and Pielou’s evenness indices, was significantly higher in the gastric tissues of the HFD and sHFD groups than in those of the NCD group (Fig. 2A, left). In the fecal samples, microbial community richness (indicated by the Shannon, Simpson’s, and Pielou’s evenness indices) was not significantly different between the three groups. However, community diversity (as estimated by the Chao 1 index) was significantly lower in the HFD and sHFD groups than in the NCD group (Fig. 2A, right, Supplementary Figure S3A, B). In the PCoA results based on Bray–Curtis distances and PERMANOVA, when compared to the NCD group, significant differences were observed in gastric and fecal samples in both the HFD and sHFD groups, respectively (Fig. 2B, Supplementary Figure S3A, B, Supplementary Table S5).

### Relative abundance of microbes in the gastric and fecal microbiomes

**Figure 3:**
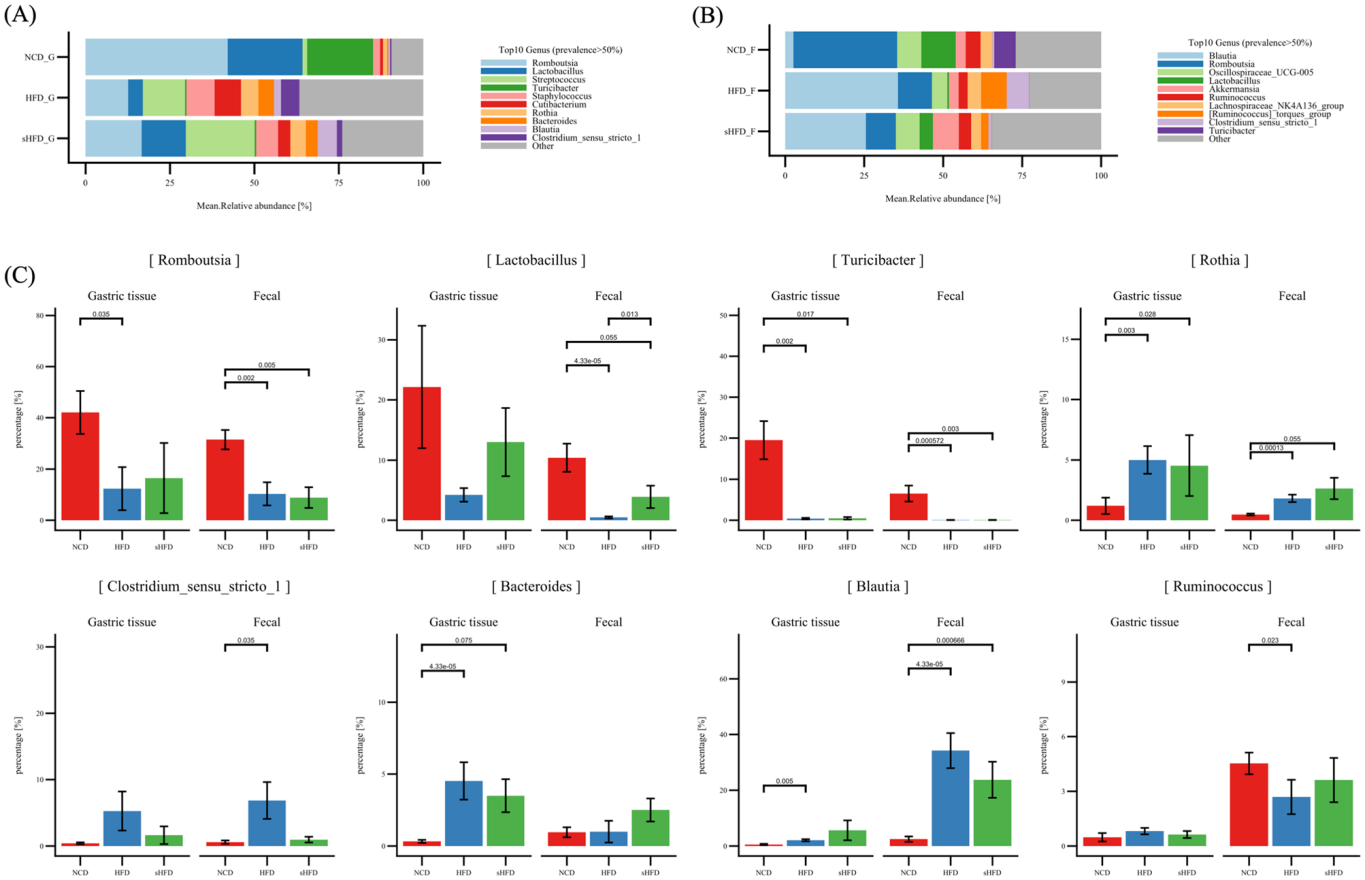
Relative abundance of dominant microbes in the gastric- and fecal-derived microbiota of the NCD, HFD, and sHFD groups. Relative abundances of microbes in (**A**) gastric and (**B**) fecal samples. (**C**) Abundance of 8 microbes that are common among the top 20 dominant genera in the gastric and fecal samples of each group. The X-axis represents each group, and the Y-axis represents the proportion (%) of each microbiota.

At the phylum level, *Firmicutes* constituted the majority of microbes (relative abundance, 87.5%) in the gastric tissues of the NCD group, followed by *Actinobacteria* (2.1%) and *Bacteroidota* (2.94%). *Firmicutes* (48%), *Actinobacteria* (11%), *Proteobacteria* (4.3%), and *Bacteroidota* (5.5%) were the major microbes in the HFD group. *Firmicutes* (57.6%), *Actinobacteria* (9.2%), *Bacteroidota* (3.39%), and *Proteobacteria* (4.1%) were most abundant in the sHFD group. The relative abundance of *Firmicutes* was lower in the HFD group than in the NCD and sHFD groups, whereas those of *Actinobacteria* and *Bacteroidota* were higher (Supplementary Figure S5A). At the genus level, *Romboutsia* (40%), *Lactobacillus* (21%), *Turicibacter* (18.8%), and *Streptococcus* (1.2%) were dominant in the NCD group. *Streptococcus* was more abundant in the HFD group (9.1%) than in the NCD group (1.2%). In the sHFD group, *Streptococcus* showed high abundance (18%), whereas *Romboutsia* (14%) and *Lactobacillus* (8.5%) were less abundant than in the NCD group (Fig. 3A, Supplementary Table S6-S7).

There were no significant between-group differences in the composition of the fecal microbiome at the phylum level. *Firmicutes* was most abundant (94%) in the NCD group, followed by *Verrucomicrobiota* (2.7%), *Bacteroidota* (1.5%), and *Actinobacteria* (0.7%) (Supplementary Figure S5B). In the HFD group, the relative abundances of *Firmicutes*, *Verrucomicrobiota*, *Bacteroidota*, and *Actinobacteria* were 92%, 2.5%, 1.64%, and 3%, respectively. The sHFD group showed high abundance of *Firmicutes* (85%), *Verrucomicrobiota* (6.9%), and *Bacteroidota* (3.6%). At the genus level, the NCD group showed high abundance of *Romboutsia* (27%) and *Lactobacillus* (9.1%). However, the dominant microbes changed dramatically in the HFD group, which showed high abundance of *Blautia* (30%) and lower abundance of *Lactobacillus* (0.4%). The sHFD group showed a similar trend to the HFD group. *Akkermansia* (6.9%) was more abundant in the sHFD group than in the HFD group (Fig. 3B, Supplementary Figure S5B, Supplementary Table S6-S7).

Our results can also be interpreted in terms of gastric versus fecal motility. *Lactobacillus* and *Romboutsia* were most abundant in the gastric and fecal samples of the NCD group. *Turicibacter* was dominant in gastric samples, whereas *UCG-005*, *Lactobacillus*, *Akkermansia*, *Ruminococcus*, *Lachnospiraceae NK4A136 group*, *Ruminococcus torque group*, *Clostridium *sensu*-stricto-1*, and *Turicibacter* were dominant in fecal samples. *Streptococcus*, *Staphylococcus*, *Cutibacterium*, *Bacterolides*, *Blautia*, and *Clostridium *sensu*-stricto-1* (microbes that frequently appear in the oral cavity) were also highly abundant in the HFD group, but *Romboutsia* and *Lactobacillus* were relatively less abundant (Fig. 3A, B). *Blautia* was most abundant in the fecal samples of the HFD group, followed by *Romboutsia*, *UCG-005*, *Akkermansia*, *Ruminococcus*, *Lachnospiraceae NK4A136 group*, *Ruminococcus torque group*, and *Clostridium *sensu*-stricto-1*. However, *Lactobacillus* was less abundant in these samples. *Streptococcus* was the most abundant microbe in the gastric samples of the sHFD group, followed by *Romboutsia* and *Lactobacillus.* In the fecal samples of the sHFD group, *Blautia* and *Romboutsia* were most abundant, followed by *Akkermansia*.

To characterize the microbial composition of gastric and fecal samples in the three experimental groups, we compared the abundances of 8 common genera among the top 20 most abundant microbes (Supplementary Figure S6). *Romboutsia*, *Lactobacillus*, and *Turicibacter* showed high abundances in the NCD group (Fig. 3C) and lower abundances in the other groups. Interestingly, the abundances of *Romboutsia* and *Lactobacillus* were higher in the gastric tissue of the sHFD group than in those of the HFD group. *Rothia*, *Clostridium *sensu*-stricto-1*, *Bacteroides*, and *Blautia* were more abundant in the HFD group than in the NCD group. In contrast, *Ruminococcus* was more abundant in the gastric tissues of the HFD group than in those of the NCD and sHFD groups, but lower in the fecal tissues of the HFD group than in those of the NCD and sHFD groups.

**Figure 4:**
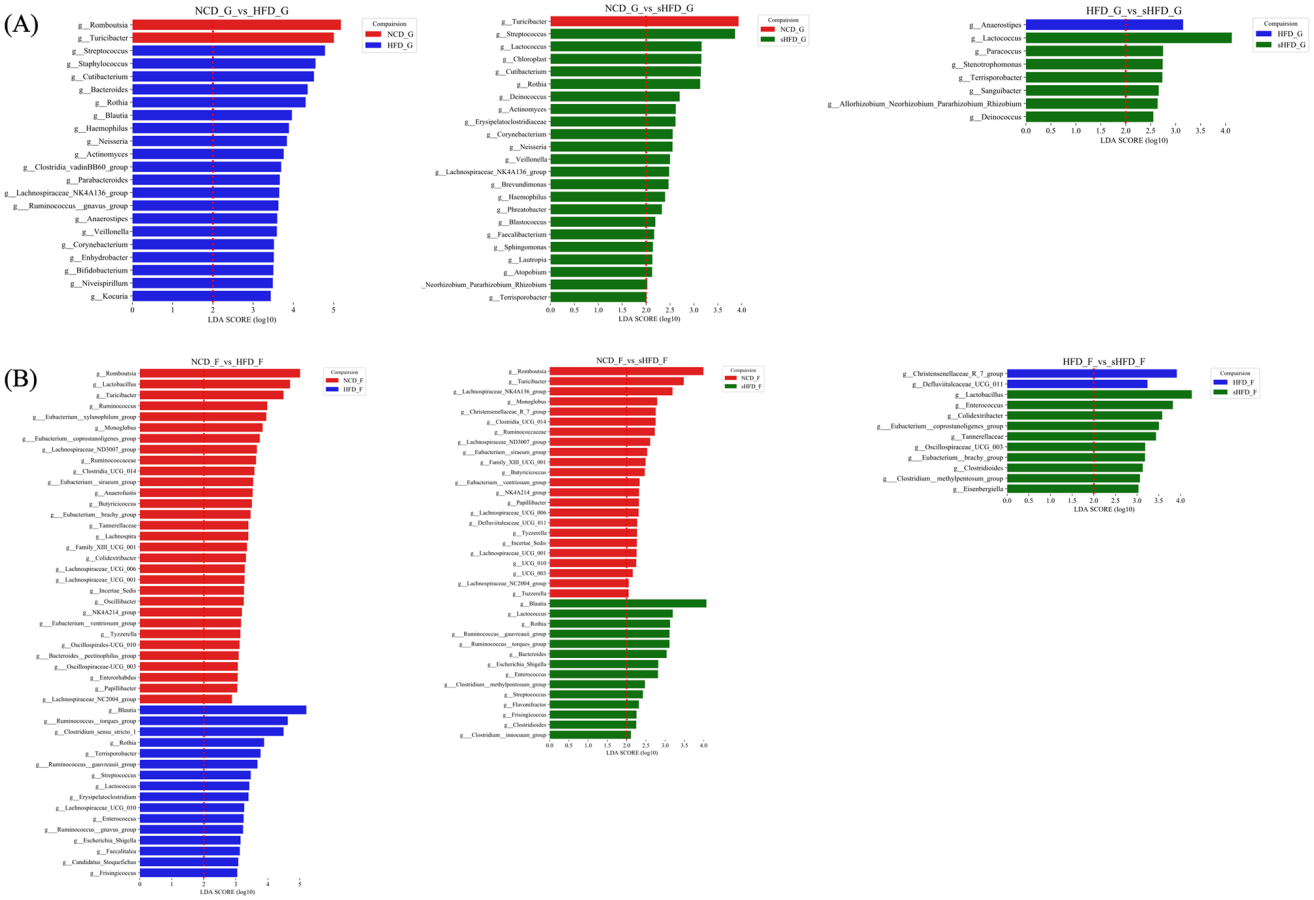
Impacts of the NCD, HFD, and sHFD treatments on the composition of gastric and fecal microbiota. LEfSe analysis of the gastric- and fecal-derived microbiota in each group. For each comparison, genera with LDA scores > 2.0 are shown. Results for NCD vs. HFD, NCD vs. sHFD, and HFD vs. sHFD are shown for (**A**) gastric and (**B**) fecal samples. NCD: Normal chow diet group, HFD: High fat diet group, sHFD: after high fat diet, gastrectomy group.

We performed LEfSe analysis to examine the extent of change in gut and fecal microbes that showed significant differences between each group (Fig. 4). *Romboutsia* and *Turicibacter* were significantly enriched in the gastric samples of the NCD group, whereas *Streptococcus, Staphylococcus, Cutibacterium*, *Bacteroides*, and *Rothia* were more abundant in the gastric samples of the HFD group. *Anaerostipes* was significantly enriched in the gastric samples of the HFD group, whereas *Lactobacillus*, *Paracoccus*, *Stenotrophomonas*, *Terrisporobacter*, *Sanguibacter*, *Allorhizobium*, and *Deinococcus* were more abundant in those of the sHFD group (Fig. 4A, Supplementary Table S8).

*Romboutsia*, *Lactobacillus*, *Turicibacter*, *Ruminococcus*, and *Xylanophilum group* were significantly enriched in the fecal samples of the NCD group, whereas *Blautia*, *Ruminococcus torques group*, *Clostridium *sensu*-stricto-1*, *Rothia*, *Terrisporobacter*, *Ruminococcus gauvreauii group*, and *Lactococcus* were more abundant (LDA > 3.0) in those of the HFD group. *Defluviitaleaceae UCG-011* and *Christensenellaceae R7 group* were significantly enriched in the fecal samples of the HFD group, whereas *Lactobacillus* and *Enterococcus* were more abundant in the fecal samples of the sHFD group (Fig. 4B, Supplementary Table S9).

### Correlations between gastric- and fecal-derived microbes and clinical parameters

**Figure 5:**
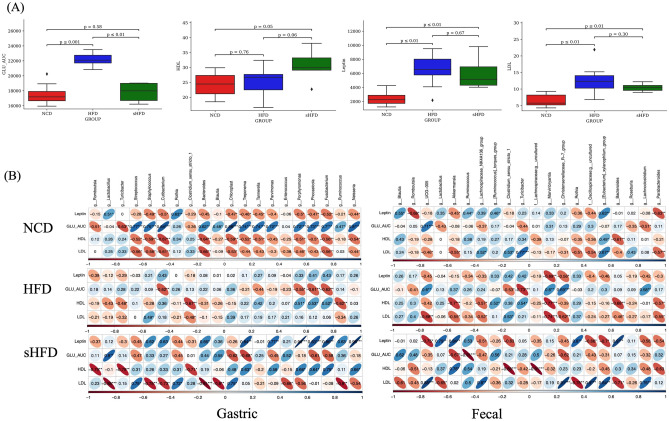
Correlation analysis between clinical parameters that were significantly different between groups (*p* < 0.05) and gut/fecal microbiome composition. (**A**) Bar plots showing significant differences in clinical parameters between groups. (**B**) Correlation matrix showing correlations between clinical parameters and the abundances of microbes. The size and color intensity of the ellipses indicate the strength of positive or negative correlation (blue color, positive correlation; red color, negative correlation). The black asterisk (* < 0.05; ** < 0.01; *** < 0.001) within the ellipses represents the p-value of the statistical test. NCD: Normal chow diet group, HFD: High fat diet group, sHFD: after high fat diet, gastrectomy group.

The levels of hormones, such as leptin and ghrelin, can change after bariatric surgery. Hormonal changes are known to be associated with metabolism and the microbiota^29^. Therefore, we performed Spearman correlation analysis to examine how the gut and fecal microbiomes of each group were correlated with clinical parameters. The top 20 most abundant genera in gastric and fecal samples and the clinical parameters that were significantly different between groups (*p* < 0.05) were used in these analyses (Fig. 5A,B, Supplementary Figure S7, Supplementary Table S10-S11).

The abundance of *Bacteroides* showed significant negative correlations with HDL levels in the NCD group (gastric: correlation coefficient [r] = − 0.64, *p* = 0.0021; fecal: r = − 0.61, *p* = 0.0041). In the HFD group, the abundance of *Turicibacter* was significantly positively correlated with HDL levels (gastric: r = − 0.480, *p* = 0.04; fecal: r = 0.538, *p* = 0.017). Moreover, LDL levels in the HFD group were correlated with the abundances of *Clostridium *sensu*-stricto-1* (r = − 0.480, *p* = 0.04) and *Rothia* (r = 0.7190, *p* = 0.013) in gastric samples. Similar results were observed for fecal samples as well (*Clostridium *sensu*-stricto-1*: r = 0.529, *p* = 0.019; *Rothia*: r = − 0.783, *p* = 0.007). In the sHFD group, both gastric-derived and fecal-derived *Clostridium *sensu*-stricto-1* showed significant correlations with HDL levels (gastric: r = − 0.71, *p* = 0.02; fecal: r = − 0.878, *p* = 0.0008). Gastric-derived *Bacteroides* and *Lactobacillus* also showed significant correlations with LDL levels (*Bacteroides*: r = − 0.90, *p* = 0.0002; *Lactobacillus*: r = − 0.87, *p* = 0.0009). Similar results were observed for fecal-derived microbes as well (*Bacteroides*: r = − 0.71, *p* = 0.02; *Lactobacillus*: r = − 0.64, *p* = 0.04). Both gastric-derived and fecal-derived *Bacteroides* showed significant correlations with leptin levels (gastric: r = 0.657, *p* = 0.038; fecal: r = 0.89, *p* = 0.00043) (Table 2).

**Table 2:** Correlations between clinical parameters and microbial abundance in gastric and fecal samples (those with significant differences at *p* < 0.05).

Group	Clinical_parameter	Taxon	G_corr^†^	G_p.value^‡^	F_corr^†^	F_p.value^‡^
NCD	Insulin	g__Lactobacillus	− 0.480320834	0.032070725	0.565115262	0.009420096
	TG	g__Rothia	0.489082979	0.028636441	− 0.530063157	0.016216839
	AST	g__Turicibacter	0.450953077	0.045977818	0.631625147	0.002815659
	BW_mean	g__Romboutsia	− 7.47E−01	0.000153386	− 0.729210635	0.000264375
	HDL	g__Bacteroides	− 0.643715039	0.002194465	− 0.612338358	0.004105842
HFD	GIP	g__Turicibacter	− 0.560968921	0.015435401	0.760687717	0.000156033
	HDL	g__Turicibacter	− 0.480969388	0.043317396	0.538447729	0.01738758
	LDL	g__Clostridium_sensu_stricto_1	− 0.480891347	0.043356289	0.529122941	0.019832064
	BW_mean	g__Rothia	0.468331759	0.049969759	0.476260717	0.039266488
	Glucagon	g__Rothia	0.485795455	0.040963493	0.582363996	0.008889153
	Insulin	g__Rothia	0.913416924	1.21E−07	0.712664411	0.000616484
	Diet_mean	g__Rothia	− 0.742214276	0.013962001	0.974868716	1.69E−06
	LDL	g__Rothia	0.719159838	0.019073792	− 0.783382278	0.00734787
	Diet_mean	g__Bacteroides	0.839751282	0.002366471	0.992844885	1.14E−08
sHFD	HDL	g__Clostridium_sensu_stricto_1	− 0.712396498	0.020789715	− 0.878768967	0.000814351
	LDL	g__Bacteroides	− 0.907939084	0.000280851	− 0.710715899	0.02123207
	LDL	g__Lactobacillus	− 0.875511836	0.000901761	− 0.649804964	0.04198073
	Leptin	g__Bacteroides	0.657722766	0.038729004	0.896666654	0.000439586

G_corr^†^ Correlation between the gastric derived microbiota and clinical parameter.

F_corr^†^ Correlation between the fecal derived microbiota and clinical parameter.

G_*p* value^‡^: Significant value between the gastric derived microbiota and clinical parameter.

F_*p* value^‡^: Significant value between the fecal derived microbiota and clinical parameter.

NCD: Normal chow diet group, HFD: High fat diet group, sHFD: after high fat diet, gastrectomy group.

### Correlations between functional metabolic pathways and clinical parameters

We performed pathway analysis using the ASVs data of respective samples to examine the correlations between clinical data and microbiome composition in each group. The analysis was conducted in three steps. First, the metabolic pathways enriched in PICRUSt2 were extracted from each group (Supplementary Figure S8-S9). Second, we extracted the pathways that were common between groups and tissue locations. Finally, we analyzed the correlation between the metabolic pathways (*p* < 0.05) and clinical parameters that were significantly increased in sHFD.

Glyoxylate and dicarboxylate metabolism (ko00063) was increased in the sHFD group (*p* < 0.05), compared with the NCD group (Table 3), and showed a negative correlation with the glucose AUC (r = − 0.58). Taurine and hypotaurine metabolism (ko00430) were increased in the sHFD group than in the HFD group (*p* < 0.05) and were negatively correlated with mean diet (weeks 10–14; r = − 0.46). Butanoate metabolism (ko00650) was increased in the sHFD group (*p* < 0.05) and was negatively correlated with the glucose AUC (r = − 0.35) and mean diet (r = − 0.56). Nitrogen metabolism (ko00910) was increased in the sHFD group, compared with that in the NCD group (*p* < 0.05), and was positively correlated with the glucose AUC (r = 0.701), leptin (r = 0.689), triglycerides (r = 0.375), LDL (r = 0.701), and mean body weight (r = 0.558). Pyrimidine metabolism (ko00240) was increased in the sHFD group, compared with the HFD group (*p* < 0.05), and was positively correlated with insulin (r = 0.434) and negatively correlated with leptin (r = − 0.666), LDL (r = − 0.578), and mean body weight (r = − 0.472). Aminoacyl-transfer RNA (tRNA) biosynthesis (ko00970) was increased in the sHFD group, compared with the HFD group (*p* < 0.05), and was positively correlated with the glucose AUC (r = − 0.766), leptin (r = − 0.741), LDL (r = − 0.671), and mean body weight (r = − 0.566) (Supplementary Table S12-S13).

**Table 3:** Correlations between clinical parameters and functional metabolic pathways (clinical parameters with |R|> 0.2 in fecal and gastric samples).

Consensus pathway of "NCD/HFD vs sHFD" and " Samczuk et al."	Description	Site	Compared group	Increased Group	Correlated clinical parameter with pathway (r)
ko00063	Glyoxylate and dicarboxylate metabolism	Fecal	NCD vs sHFD	sHFD	Glu_AUC (− 0.58)
ko00430	Taurine and hypotaurine metabolism	Fecal	HFD vs sHFD	sHFD	Diet_mean (− 0.46)
ko00650	Butanoate metabolism	Fecal	HFD vs sHFD	sHFD	Glu_AUC (− 0.35)
					Diet_mean (− 0.56)
ko00910	Nitrogen metabolism	Fecal	NCD vs sHFD	sHFD	Glu_AUC (0.701)
					Leptin (0.689)
					TG (0. 375)
					LDL (0.701)
					BW_mean (0.558)
ko00240	Pyrimidine metabolism	Fecal	HFD vs sHFD	sHFD	Glu_AUC (− 0.739)
					Insulin (0.434)
					Leptin (− 0.666))
					LDL (− 0.578)
					BW_mean (− 0.472)
ko00970	Aminoacyl-tRNA biosynthesis	Fecal	HFD vs sHFD	sHFD	Glu_AUC (− 0.766)
					Leptin (− 0.741)
					LDL (− 0.671)
					BW_mean (− 0.566)

NCD: Normal chow diet group, HFD: High fat diet group, sHFD: after high fat diet, gastrectomy group.

Liquid chromatography-mass spectrometry and nuclear magnetic resonance are the most frequently used techniques to study the main effects of Roux-en-Y or SG. Samczuk et al. reviewed how metabolites and metabolite pathways were affected by various bariatric surgeries. The metabolite pathways reported by Samczuk et al. overlap with those associated with changes in the microbial composition of the sHFD group in this study^30^. Daniel et al. reported that porphyrin and chlorophyll metabolism and fatty acid biosynthesis were associated with HFD-specific metabolism, and that urobilinogen—a metabolite produced by microbes that is involved in porphyrin and chlorophyll metabolism—was specific to HFD (Daniel et al., 2014). This is consistent with our results, as fatty acid biosynthesis was higher in gastric-derived NCD samples and fecal-derived sHFD samples. However, this was inconsistent with the results of fatty acid biosynthesis (*p* = 0.23) reported by Samczuk et al. (Supplementary Figure S10**,** Supplementary Table S14).

### Co-occurrence of gastric- and fecal-derived microbes across groups

We performed co-occurrence analysis to predict connectivity between the gastric- and fecal-derived microbiota. In gastric tissues, the NCD group showed relatively lower rates of co-occurrence than the HFD and sHFD groups. The HFD group showed more interactions than the NCD group and fewer interactions than the sHFD group. The sHFD group showed a strong interaction overall. This result may indicate that a high-fat diet increases the diversity of microbes in a limited environment (such as pH and gastric wall thickness, which are characteristic of certain organs).

**Figure 6:**
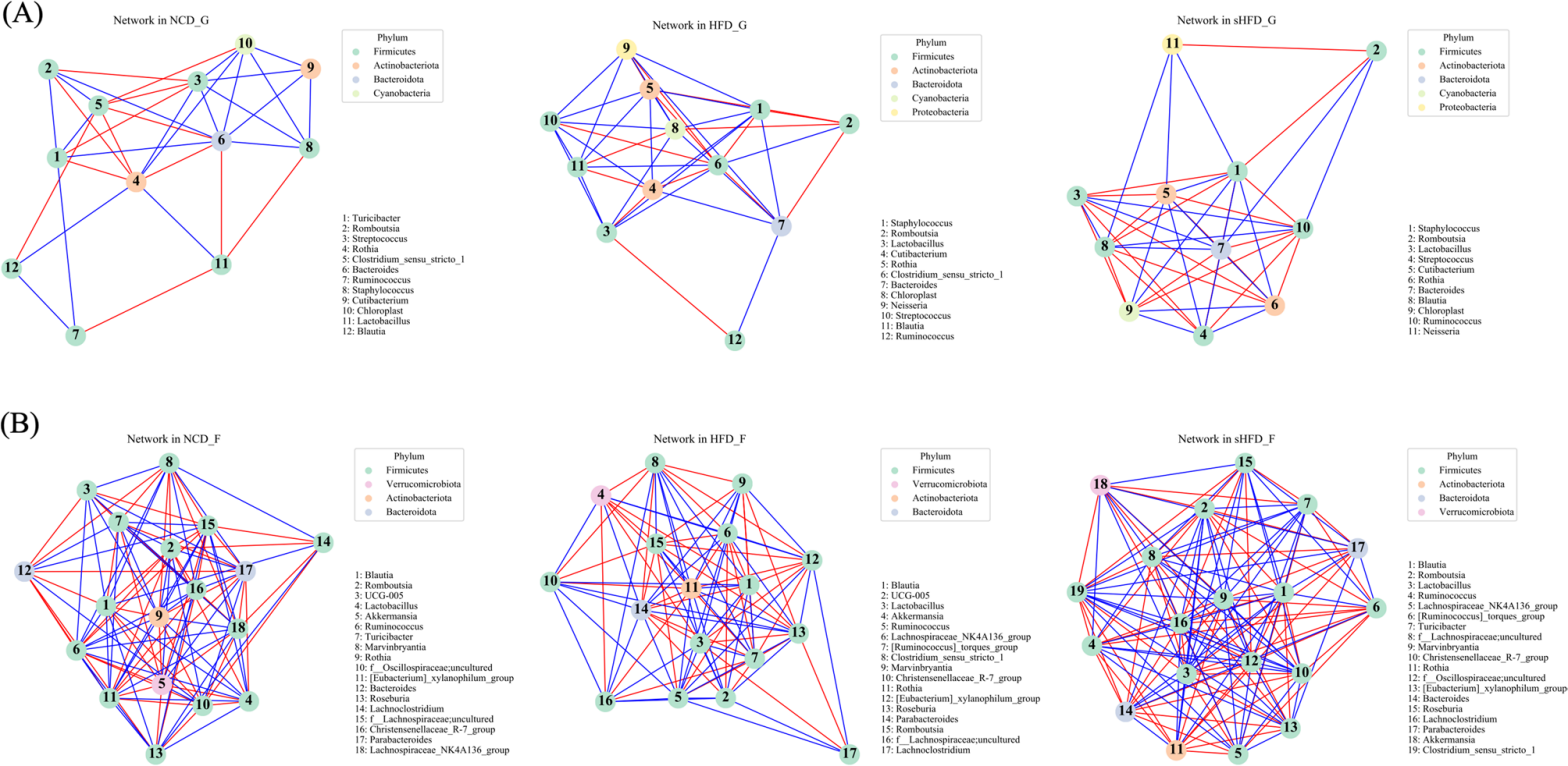
Co-occurrence networks of the top 20 microbial genera in the gastric and fecal samples of the NCD, HFD, and sHFD groups. The top 20 genera were identified based on SparCC values. Co-occurrence networks are shown for (**A**) gastric and (**B**) fecal samples. The nodes represent the microbial genera, and the line colors indicate negative (red) or positive (blue) correlation. NCD_G: Gastric derived microbiota in normal chow diet group, HFD_G: Gastric derived microbiota in high-fat diet group, sHFD_G: Gastric derived microbiota in HFD-induced obese rats that underwent sleeve gastrectomy group. NCD_F: Fecal derived microbiota in normal chow diet group, HFD_F: Fecal derived microbiota in high-fat diet group, sHFD_F: Fecal derived microbiota in HFD-induced obese rats that underwent sleeve gastrectomy group.

**Figure 7:**
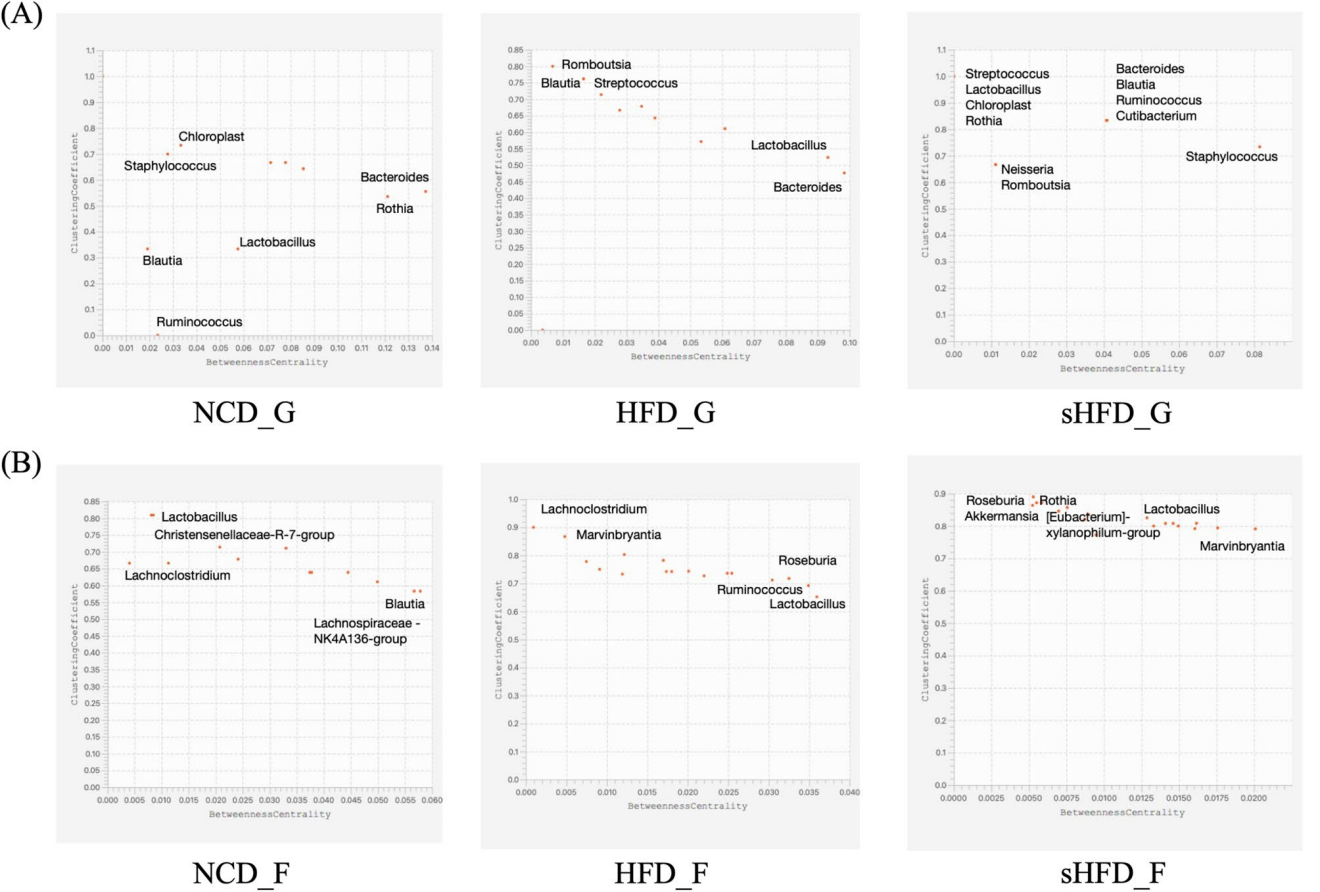
Co-occurrence between microbes based on betweenness centrality (BNC) and clustering coefficient (CC). Each plot shows the intersection between BNC and CC values. Only most prominent genera of bacteria are included. The X-axis is BNC (Betweenness Centrality) and the Y-axis is CC (Clustering Coefficient). Microbial genera located at the bottom right of the plot have the lowest co-occurrence rates compared to other genera (low CC), meaning they are at the center of the network topology (high BNC).

In the fecal tissues, microbial co-occurrences in the NCD group were more interactive than those in the HFD group. The HFD group showed fewer interactions than other groups, likely due to the effects of dysbiosis. The sHFD group showed stronger co-occurrence and interactions (Fig. 6A, B, Supplementary Figure S11A, B, Supplementary Table S15). The patterns of interaction were compared using the BNC and CC. In Fig. 7, genera located in the bottom-right of the plots showed the lowest rates of co-occurrence with other genera (low CC) and were central in the topology of the network (high BNC). Thus, microbes that were central to the network had the lowest rates of co-occurrence with other genera (Fig. 7).

In gastric tissues, *Bacteroides* showed the highest BNC in the NCD group, followed by *Rothia*. *Chloroplast* and *Staphylococcus* showed high CC in the NCD group. In the HFD group, *Bacteroides* showed the highest BNC, followed by *Lactobacillus*. In the sHFD group, *Staphylococcus* showed the highest BNC, and *Bacteroides*, *Blautia*, *Ruminococcus*, and *Cutibacterium* showed high CC. In fecal tissues, *Blautia* and *Lachnoclostridium* had the highest BNC in the NCD group, whereas *Christensenellaceae R7 group* and *Lactobacillus* had the highest CC. *Lactobacillus* showed the highest BNC in the HFD group, whereas *Lachnoclostridium* and *Marvinbryantia* had the highest CC values. *Marvinbryantia* showed the highest BNC in the sHFD group, and CC values were highest in *Roseburia*, *Rothia*, *Eubacterium xylanophilum group*, and *Akkermansia*.

Overall, BNC values showed a wider range in gastric tissues than in fecal tissues. In contrast, the CC values of microbes showed a narrower range ​​in fecal tissues than in gastric tissues. This indicates that core microbes in fecal tissues (rather than gastric tissues) act through direct connection rather than through interactions with neighboring microbes. Core microbes are thought to act through direct and indirect connectivity in gastric tissues with relatively low microbial diversity. *Streptococcus*, *Lactobacillus*, *Chloroplast*, and *Rothia* showed a CC value of 1 and all microbes, except *Neisseria* and *Romboutsia*, showed CC values of ≥ 0.7 in gastric tissues. Likewise, most microbes had a CC of ≥ 0.7 in fecal tissues. Interestingly, CC values were generally higher in the HFD and sHFD groups than in the NCD group. The CC value in each group increased when 0.7 was set as the cutoff. When comparing BNC values with a cutoff of 0.01, the number of microbes with BNC values higher than the cutoff gradually decreased.

## Discussion

Rossell et al. reported that following diet control and SG, the body weight, obesity index, and the levels of leptin, ghrelin, and insulin sensitivity index returned to baseline control values in the high-fat diet-induced obesity group^29^. Consistent with this, we found that leptin levels, body weight, fasting glucose, HDL, and LDL levels showed similar patterns in our experimental groups. As such, these results seemed to be the positive outcomes of SG.

Regarding the changes in microbiota, He et al. showed that the microbial changes in the gastric microbiome after a HFD diet were significantly different from those in the NCD group. For 12 weeks, the HFD-fed mouse model reported a decrease in α-diversity and no change in fecal microbiome compared to the NCD-fed model. In addition, the relative α-diversity in the HFD group was high in the 24-week diet model. Although it was not significant in the fecal microbiome, relatively decreased α-diversity was shown^31^. This result shows a similar pattern to the α-diversity in the stomach of the 14-week dietary model in our data. As a result, there was a significant increase and, moreover, it was confirmed that the diversity was increased even in the model in which gastrectomy was performed after HFD induction. On the other hand, the fecal microbiome of the HFD group showed a significantly decreasing pattern. After sleeve gastrectomy, the HFD increased compared to the HFD group, although it was not significant. The 14-week model of our study was similar to the 24-week pattern of the long-term HFD model of He et al.’s study, and it was confirmed that the microbiota pattern after gastric resection showed a recovery pattern like that of the NCD group. The microbiota pattern is thought to be less likely to change in the gut because of the stronger resistance of the predominant bacterial community and the stronger ability to self-regulate. On the other hand, the structure of the above microorganisms is relatively simple; therefore, it is thought to be more sensitive^32^.

High-fat diet-induced obese rodents show an altered microbial community set compared to control animals^33^. Moreover, obese animals show low microbial diversity and altered abundance of the major intestinal phyla *Firmicutes* and *Bacteroidetes*^34–36^. Reduced bacterial abundance is associated with conditions such as insulin resistance, dyslipidemia, and obesity^37^. In other words, it is known to cause an “unbalanced” condition that causes an imbalance in the gut microbiota. Therefore, the increase in bacterial abundance following sleeve gastrectomy in our study may reflect a positive effect on dysbiosis in high-fat diet-induced obese rodents.

Overall, the composition of the gastric microbiome was considerably different in the HFD and sHFD groups than in the NCD group. Intriguingly, the diversity of the gastric microbiota was retained despite changes in the gastric pH, mucosal thickness, and peristalsis, which are known to limit microbial growth. *Firmicutes*, *Bacteroidetes*, *Fusobacteria*, *Actinobacteria*, and *Proteobacteria* were more abundant at the phylum level, whereas *Prevotella, Streptococcus, Veillonella, Rothia*, and *Haemophilus* were more abundant at the genus level^38^. This pattern of microbial composition is similar to that seen in conditions of functional dyspepsia, which is caused by a high-fat diet. In particular, functional dyspepsia is strongly associated with an increase in the abundance of *Streptococcus* in the gastrointestinal tract. This may explain the increased abundance of *Streptococcus* in the high-fat diet groups in our study^39,40^.

In the case of *Akkermansia muciniphila*, it has been considered as an important functional microorganism with probiotic properties in host metabolism^41^. Another study also reported that an increase in *Akkermansia* recovered the HFD-induced intestinal dysbiosis and improved obesity^42^. In our results, *Akkermansia* spp. was not detected in the gastric microbiome, but was detected in the fecal microbiome. Also, the relative abundance decreased in the HFD diet-induced obesity model, but increased in the group after sleeve gastrectomy.

In other studies, HFDs exhibit distinct dysbiosis, particularly characterized by a significant decrease in *Lactobacillus* abundance. At the same time, transplantation of the proximal small intestine microbiome from rats fed a low-fat diet into the duodenum of rats fed a HFD was shown to partially restore normal abundance of *Lactobacillus* and reduce metabolic disturbances induced by HFD ingestion^43^. It was also reported that the increase in lactic acid caused by the increase of lactobacillus in the duodenum by vertical sleeve gastrectomy induces HIF activation and thus induces various positive metabolisms^44^.

Several studies evaluated metabolite-based changes after HFD studies and bariatric surgery^29,45,46^. However, few comparative studies have investigated the aforementioned microbes. Rossell et al. investigated the effects of HFD on gastric and intestinal microflora after varying periods of time. The results showed that the composition of the gastric microbiota changed earlier than that of the gut microbiota, indicating that the gastric microbiota are more sensitive to HFD^31^. Our results are consistent with these findings.

In addition, six common metabolic pathways were detected from the results of our predicted microbial metabolic pathways and those identified in the serum by Samczuk et al. These metabolic pathways suggest that microbial-derived metabolic pathways are important to the host after surgery.

Cooccurrence data showed that some specific core microbiota in the interaction between microorganisms differed in the gastric and gut samples of HFD-induction, as well as after surgery. This was confirmed through the CC^47^ and BNC^48,49^. The clustering coefficient defines how nodes are connected in their neighbors. And the betweenness centrality is defined as the degree to which this node controls other nodes.

In our results, *Lactobacillus* in the stomach had a higher CC in the HFD group. This result means that the connection between *Lactobacillus* and other neighboring nodes was the highest. However, after gastrectomy, *Lactobacillus* showed the opposite result and BNC was higher. This could be due to the effect of *Lactobacillus* on the network of other microorganisms. In other words, *Lactobacillus* could be explained as the connecting core of the other microbiota. In the fecal microbiome, the BNC could be explained as the connecting core of *Roseburia*, *Rothia*, and *Akkermansia* about the other microbiota.

Consequently, in metabolic disorders such as obesity, physical changes by SG provide for a reconstruction of the intestinal microbiota. Therefore, it is suggested that changes in microorganisms may affect positive changes in the host.

## Conclusion

In this study, we predicted significant changes in the gastric- and gut (fecal)-derived microbiota of rats in three groups treated with different dietary regimes and surgical treatments. We also analyzed how these changes were associated with clinical parameters and evaluated motility by comparing the gastric- and gut-derived microbes within each group.

Glucose AUC, leptin, and LDL were significantly different between the experimental groups. *Bacteroides*, *Turicibacter*, *Rothia*, *Clostridium *sensu*-stricto-1*, and *Lactobacillu*s were significantly correlated with these clinical parameters. Glyoxylate and dicarboxylate, taurine and hypotaurine, butanoate, nitrogen, and pyrimidine metabolism and aminoacyl-tRNA biosynthesis were affected by bariatric surgery and were significantly associated with changes in microbiome composition. Although we did not investigate the direct relationships between metabolic pathways and clinical indicators, our results suggested that gastrectomy improves obesity by affecting these metabolic pathways. Finally, we found that patterns of co-occurrence of microbes differed between groups. The groups showed increased connectivity between neighboring microbes rather than between core microbes, and co-occurrence was more prominent in fecal samples than in gastric samples.

The results of this study support the results of previous studies that profiled the gut microbiome of various HFD models. Here, we used a novel approach to characterize the gastric- and fecal-derived microbiomes of high-fat diet-induced obese rodents after gastrectomy. Although this study was conducted based only on 16 s data, there was a limit to the analysis data, and we did not perform metabolite analysis, our results elucidated the positive effects of SG in obesity. We also provided insight into alterations of the microbiota in gastric and fecal samples.

## Materials and methods

### Animals experiments

Male Sprague–Dawley (SD) rats aged 4–6 weeks were purchased from Orient Bio Inc. (Sungnam, Korea) and housed at the Biomedical Research Institute of Seoul National University Hospital. The rats were randomly assigned to three groups fed the following diets: NCD, HFD, and sHFD (Supplementary Table S1). According to the previous literature, when analyzing the gut microbiome of rats on a high-fat diet (HFD) and on a normal chow diet (NCD), the proportion of Lactobacillus was approximately 35% (with a standard deviation of 20%) in the NCD group, and about 1% in the HFD group. Based on this, assuming a power of 0.8 and α of 0.05, 5 rats per group are required. To ensure experimental reproducibility, we planned a total of 30 rats, with 10 in the control group and 20 in the experimental group (10 HFD/10 sHFD^50^. The total experimental period lasted for 14 weeks, consisting of 8 weeks of normal chow and high-fat diet feeding. After this initial period, sleeve gastrectomy and sham surgery were performed, and then, 6 weeks later, tissue and fecal samples were collected for microbiome analysis. Rats in the NCD group were fed standard rat chow (Purina rat and mouse chow, Purina Korea, Seoul, Korea) ad libitum, whereas those in the HFD and sHFD groups were fed a 60% high-fat diet (Central Lab Animal Inc., Seoul, Korea) ad libitum. After 6 weeks^51–53^, the rats in the NCD and HFD groups underwent sham operations, and those in the sHFD group underwent SG. However, 5 rats from the sleeve gastrectomy group died and were excluded from the study. The sham surgery was a laparotomy to expose the intestines, stomach and esophagus. And the abdominal wall was closed afterwards. The operative time was prolonged to mimic the degree of surgical and anesthetic stress between all groups. For rats in the sham group, laparotomy was performed to expose the stomach, esophagus, and small intestine. No other procedure was carried out. Furthermore, operative time was prolonged to induce a comparable degree of anesthetic stress as experienced by the operated rats. The role of sham group was to eliminate the influence of surgical stress anesthesia on experiments. After surgery, all rats were fed the same diet as the one before surgery^54–56^. At 6 weeks after surgery, an oral glucose tolerance test (OGTT) was performed and blood was sampled. All animal experiments were approved by the Institutional Animal Care and Use Committee of Seoul National University (approval no. 13–0273). All methods were performed in accordance with the relevant guidelines and regulations. Also, the study is reported in accordance with ARRIVE guidelines.

### Surgical techniques

After an overnight fast, either a sham operation or SG (Sleeve Gastrectomy) was performed under general anesthesia with 2% isoflurane. Ceftriaxone (50 mg/kg) was injected intramuscularly immediately before laparotomy as a prophylactic antibiotic. Normal saline (50 mL/kg) was administered subcutaneously before and after the surgery for hydration. After midline laparotomy, the greater curvature side and fundus area of the stomach were dissected. SG was performed using an Endo GIA™ Universal Stapler (Medtronic, MN, USA), and the stapling line was reinforced using 4–0 vicryl. For the sham operation, the midline laparotomy was made and closed without any resection of the intra-abdominal organs. Meloxicam (1.5 mg/kg) was administered subcutaneously for postoperative pain control. No oral intake was allowed for 24 h after the surgery, following which liquid meals and water were provided. From postoperative day 3, NCD or HFD and water were provided ad libitum. The body weight and food and water intakes of rats were monitored daily. The statistical analysis of the clinical monitoring results was conducted using Pearson correlation analysis, and the analysis was performed using R software.

### OGTT and blood sampling

The plasma concentrations of insulin, glucagon, total glucose-dependent insulinotropic polypeptide (GIP), and peptide YY (PYY) were measured using the Milliplex MAP Rat Metabolic Magnetic Bead Panel Kit (RMHMG-84 K, Millipore, Billerica, MA). The levels of other clinical markers, such as aspartate transferase (AST), alanine transaminase (ALT), low-density lipoprotein (LDL), and high-density lipoprotein (HDL), were also measured (Supplementary Table S2).

OGTTs were conducted at 6 weeks after the operation. The rats were fasted overnight and two baseline blood samples were collected. A 20% dextrose solution (1 g/kg) was administered by oral gavage, and blood samples were drawn at 15, 30, 60, 90, and 120 min after the injection. The homeostasis model assessment of insulin resistance value was calculated using the fasting glucose level and insulin concentration. All blood samples were mixed with ethylenediaminetetraacetic acid. The tubes were immediately placed on ice and centrifuged at 1500 × g for 20 min at 4 °C, and the plasma was stored at − 80 °C until further analysis (Supplementary Table S3-S4).

### Microbial genomic deoxyribonucleic acid (DNA) extraction from the gastric and fecal microbiome of rats

Animals were euthanized at 14 weeks. We used the carbon dioxide (CO2) inhalation technique for euthanasia of laboratory rats. Fresh fecal and gastric mucosa samples were collected from each group, snap-frozen in liquid nitrogen, and stored at − 20 °C. Total microbial metagenomic DNA from each sample was extracted using the QIAamp DNA microbiome kit (Qiagen, Hilden, Germany) and the experiment was carried out in accordance with the protocol of the DNA extraction kit. The quality of the extracted genomic DNA was checked using a bioanalyzer (Agilent 2100, Agilent Technologies, Inc., Santa Clara, CA, USA) and stored at − 20 °C until analysis. The Illumina platform targeted an area containing the V3-V4 hyper-variable region of the bacterial 16S rRNA gene. The PCR amplification of the target region was started immediately after the mDNA was extracted. The 16S V3-V4 amplicon was amplified using KAPA HiFi Hot Start Ready Mix (2 ×) (Roche, Penzberg, Germany). For this purpose, a pair of V3-V4 target-specific universal primers recommended by Illumina were used. The primer sequences were as follows: 16S 341F forward primer is 5′-TCGTCGGCAGCGTCAGATGTGTATAAGAGACAGCCTACGGGNGGCWGCAG-3′ and 16S 806Rreverse primer is 5′ -GTCTCGTGGGCTCGGAGATGTGTATAAGAGACAGGACTACHVGGGTATCTAATCC-3′. After the PCR amplification, the clean-up process of all PCR products was conducted using the AMPure XP beads (Beckman Coulter, California, USA). Then, additional PCR amplification was performed to add multiplexing indices and Illumina sequencing adapters using the Nextera XT Index Kit (Illumina, San Diego, CA, USA). The final PCR products were then purified once again using AMPure XP beads. After the amplicon library construction, the 16S metagenome sequencing was carried out using the paired-end 2 × 300 bp Illumina MiSeq protocol (Illumina MiSeq, San Diego, CA, USA).

### Bioinformatic analysis

The raw sequencing data generated by a MiSeq sequencer (Illumina Inc., San Diego, CA, USA) were processed using the plugins provided in the Quantitative Insights into Microbial Ecology version 2 (QIIME 2, version 2020.11) pipeline^57^. Quality-controlled amplicon sequences were corrected and PhiX-filtered, and dereplication was verified using the Divisive Amplicon Denoising Algorithm 2 plugin (DADA2) in QIIME 2^58^, whereby bacterial amplicon sequence variants (ASVs) were identified. The ASVs were aligned and a phylogenetic tree was generated using the align-to-tree-mafft-fasttree plugin in QIIME 2. Various α-diversity indices (observed features, Chao 1 index, Shannon's index, Simpson's index, and Pielou's Evenness) and β-diversity indices (Bray–Curtis and unweighted UniFrac) were determined with a rarefied depth of 1980 reads per sample by using the diversity plugin in QIIME 2. Principal coordinate analysis (PCoA) was used to investigate the similarities between bacterial communities based on metadata using the Bray–Curtis and unweighted UniFrac methods. Bacterial taxonomic assignments were performed using the SILVA (version 138, Ribocon, Bremen, Germany) 99% 16S ribosomal ribonucleic acid (RNA) database, specifically for the V3–V4 hypervariable region of 16S sequences. All classifications were implemented using the feature-classifier classify-sklearn plugin in QIIME 2.

### Statistical analysis

The Kruskal–Wallis or Mann–Whitney (Wilcoxon rank sum) tests were performed to determine significant differences in the α-diversity indices of taxonomic groups. To estimate significant differences in the β-diversity metrics of bacterial communities, permutational multivariate analysis of variance (PERMANOVA) was applied to the Bray–Curtis and unweighted UniFrac distance matrices using the ‘Adonis’ function (999 permutations) of the vegan package in R statistical software (R Core Team, Vienna, Austria). The linear discriminant analysis effect size (LEfSe) method^59^ was used to identify the taxonomical biomarker contributing to the groups with high stringency (linear discriminant analysis [LDA] score ≥ 2.0, *p*-value < 0.1). Significant biomarkers were extracted at the genus and species levels, and features marked “unclassified” were filtered out.

### Correlation analysis

Correlations between clinical parameters and the microbiome were visualized through a correlation matrix prepared using the Pearson correlation method, using the 'rcorr' function in the Hmisc package of R software. Correlation plots were implemented via the 'corrplot' function in the corrplot package of R software.

Following normalization of the Kyoto Encyclopedia of Genes and Genome (KEGG)^60–62^ pathways identified through pathway analysis (see the next section), correlations between clinical parameters and the KEGG pathways were analyzed using the Pearson correlation method.

### Metabolic pathway analysis

The Phylogenetic Investigation of Communities by Reconstruction of Unobserved States 2 (PICRUSt2) pipeline^63^ was used to infer the metagenomic functional content based on microbial community profiles obtained from the representative sequences and sequence features. Predicted functional genes were categorized using the KEGG ontology and pathway analysis. LEfSe analysis was used to evaluate differential functional abundance based on the predicted KEGG pathways.

### Co-occurrence network analysis

The ASVs table, collapsed at the genus level, was grouped by sampling site and condition. The dominant top 20 microbiomes in each sampling site were selected and, using q2-SCNIC of SparCC (Alm Lab MIT, USA), a minimal correlation limit was applied to determine the co-occurrence between them. Following this, sparse correlations for compositional data (SparCC, |R|> 0.2) were calculated using the Sparse Cooccurrence Network Investigation for Compositional (SCNIC) data plugin in QIIME2. Visualization was performed through the NetworkX (Los Alamos National Laboratory, NM, USA) of the Python library.

Betweenness centrality (BNC) and the clustering coefficient (CC) were calculated using the ‘NetworkAnalyzer’ method in Cytoscape (Cytoscape Team, CA, USA). The BNC of a node reflects the amount of control that this node exerts over the interactions of other nodes in the network^48,49^. The CC measures how well the nodes neighboring the node are connected^47^.

### Ethical approval

All animal experiments were approved by the Institutional Animal Care and Use Committee of Seoul National University (approval no. 13–0273).

### Supplementary Information

Supplementary Figures.

Supplementary Tables.

## Data Availability

The 16S rRNA gene sequences data and the metagenomic sequences data that supported the findings of this study are publicly available at the NIH National Center for Biotechnology Information Sequence Read Archive (SRA) with BioProject ID PRJNA885527.
