# Supplementary material for: Alterations in gastric and gut microbiota following sleeve gastrectomy in high-fat diet-induced obese rats
Source: Sci Rep. 2023 Dec 2;13:21294. doi: 10.1038/s41598-023-48718-w (PMC10693561; doi:10.1038/s41598-023-48718-w)
Supplement: Supplementary file 1 — Supplementary Figures. [file 41598_2023_48718_MOESM1_ESM.docx]

Supplementary Figures

## Supplementary Figures


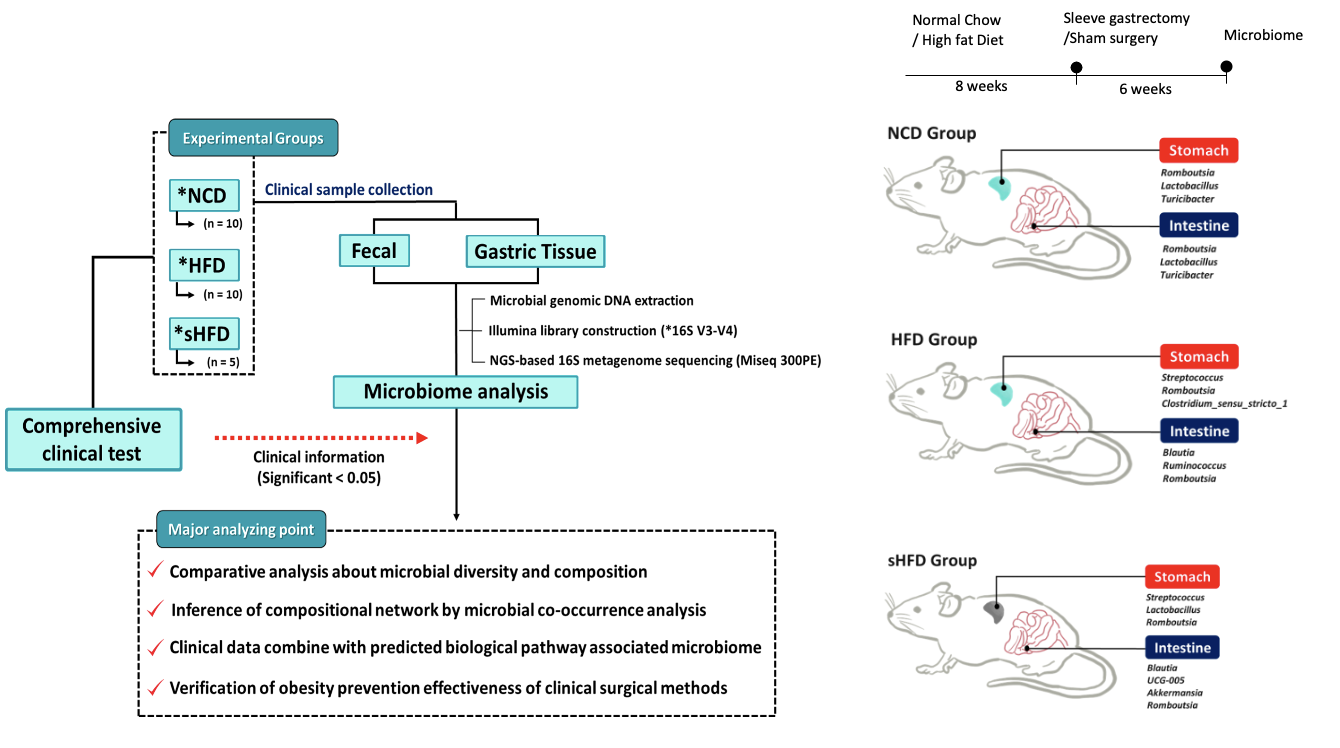


**Supplementary Figure S1.** An overall diagram for the study of Sleeve Gastrectomy in diet-induced obese rats and its impact on the microbiome.

**Supplementary Figure S2.** The alteration of clinical parameters in NCD, HFD-induced and sleeve gastrectomy rats model group


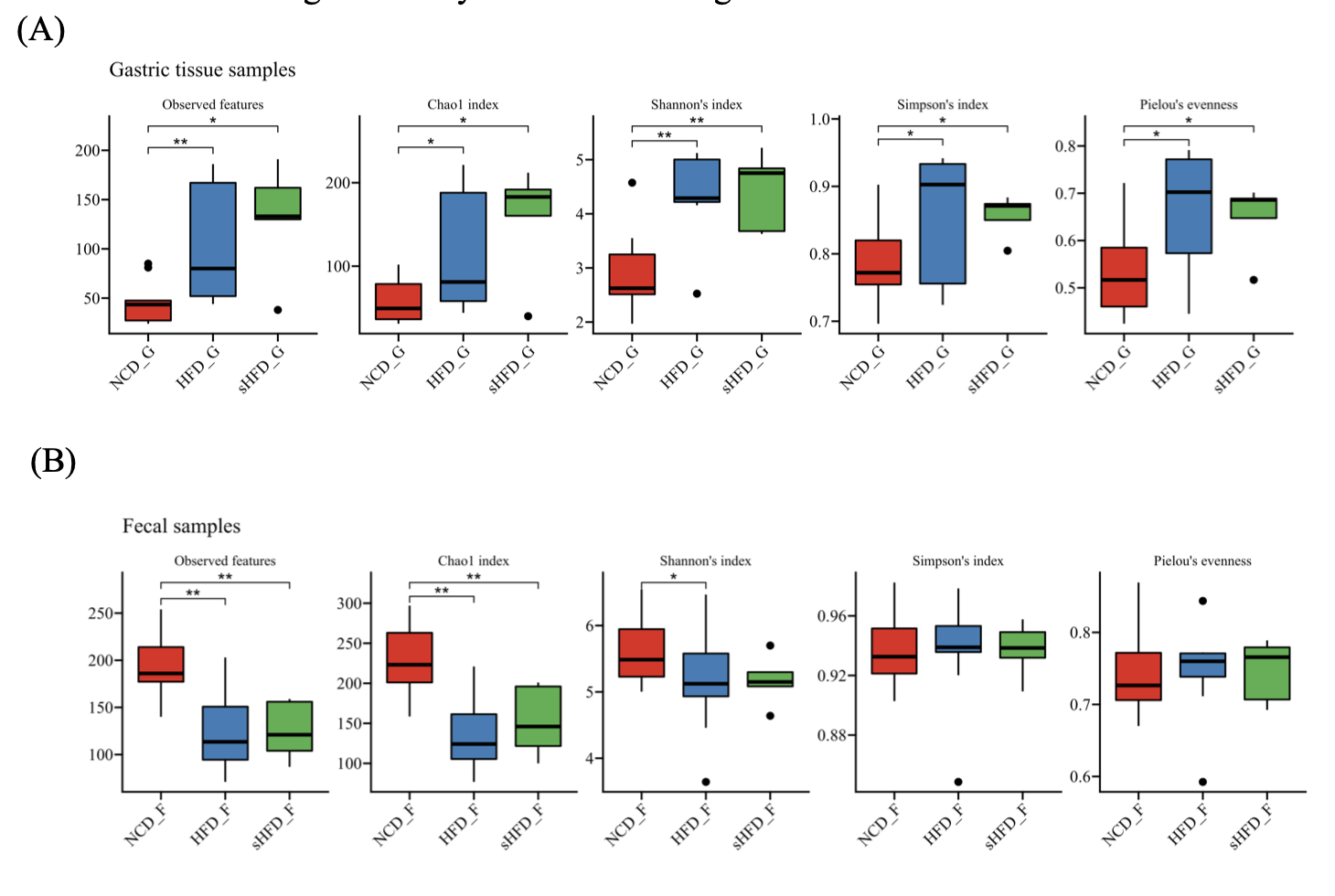


**Supplementary Figure S3.** The alpha diversity of microbiota derived from NCD, HFD-induced and sleeve gastrectomy rat model in gastric and fecal

Alpha diversity indices are shown for the gastric (A)- and fecal (B)-derived microbiota of rats fed the normal chow diet (NCD), rats fed a high-fat diet (HFD), and HFD-induced rats that underwent sleeve gastrectomy.


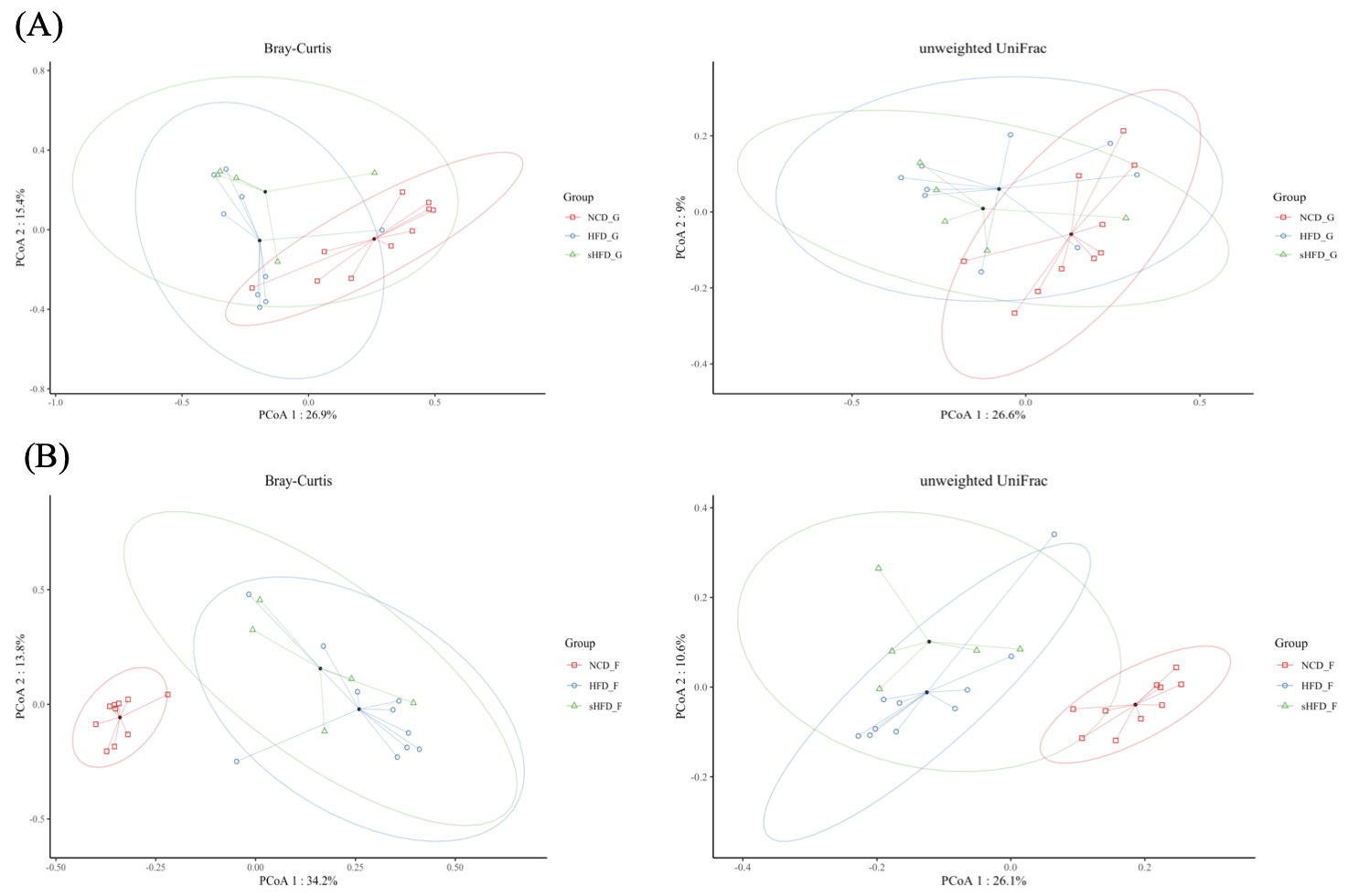


**Supplementary Figure S4.** The beta diversity of microbiota derived from NCD, HFD-induced and sleeve gastrectomy rat model in gastric and fecal

Beta diversity indices are shown for the gastric (A)- and fecal(B)-derived microbiota of rats fed the normal chow diet (NCD), rats fed a high-fat diet (HFD), and HFD-induced rats that underwent sleeve gastrectomy.




**(A)**

**
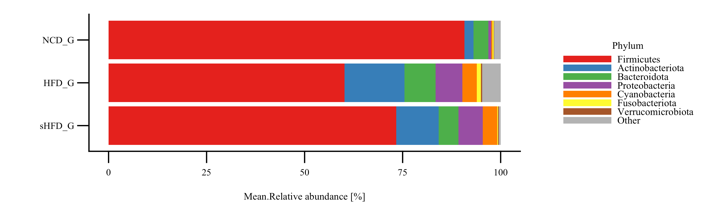

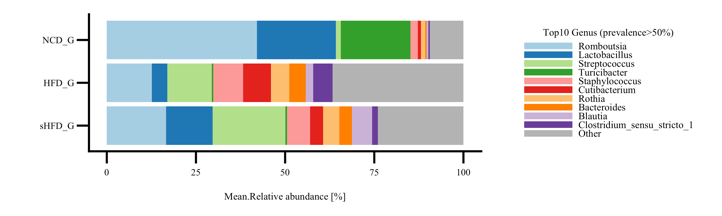
**

**(B)**

**
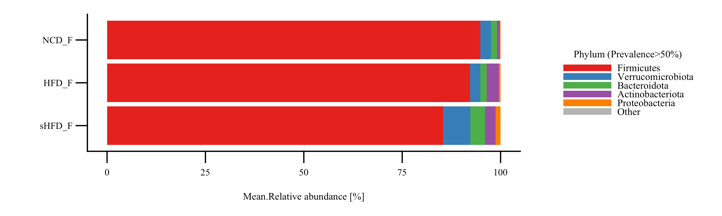

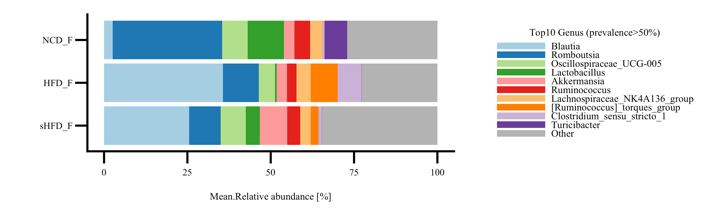
**

**Supplementary Figure S5.** The relative abundance of dominantly common microbiota in condition and sample site

Relative abundances of microbes in (A) gastric and (B) fecal samples. The left and right side indicates phylum level and genus level, respectively.

**(A) (B)**

**
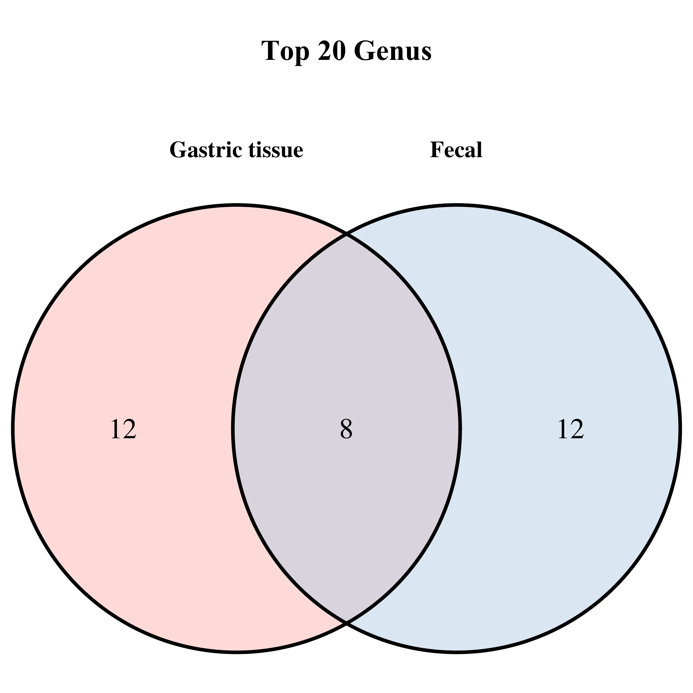

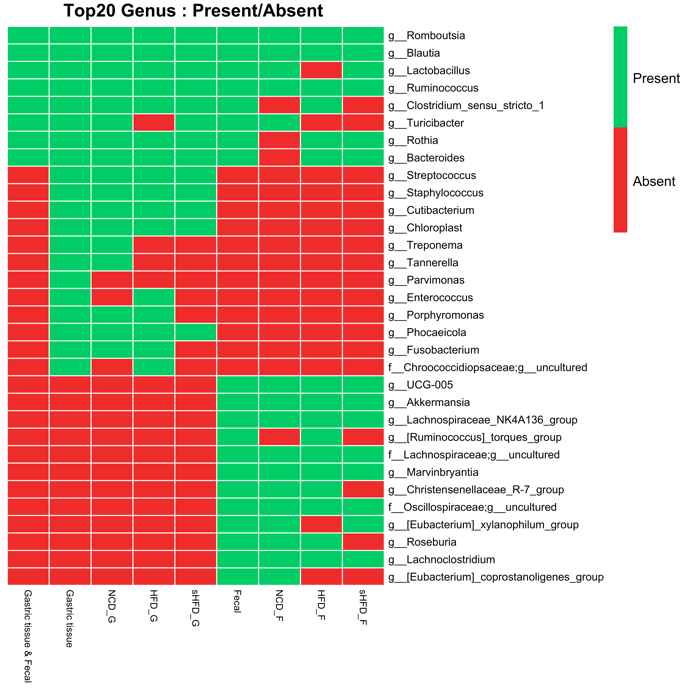
**

**Supplementary Figure S6.** **Presence of common microbiota among the top 20 genera between the gastric and fecal**

**(A) The van diagram and heatmap about dominantly top 20 genus between the gastric and fecal by group.**

**(B) Incidence within each dietary group of 8 genus common in (A)**

**(A)**

**(B)**

**Supplementary Figure S7.** **Correlation analysis between clinical parameters that were significantly different between groups (p <0.05) and gut/fecal microbiome composition**

Correlation plots are shown for the gastric (A)- and fecal(B)-derived microbiota of rats fed the normal chow diet (NCD), rats fed a high-fat diet (HFD), and HFD-induced rats that underwent sleeve gastrectomy.

**Supplementary Figure S8.** The LEfSe bar about impact of metabolic pathways between the group pair within gastric tissue

**Supplementary Figure S9** The LEfSe bar about impact of metabolic pathways between the group pair within in fecal


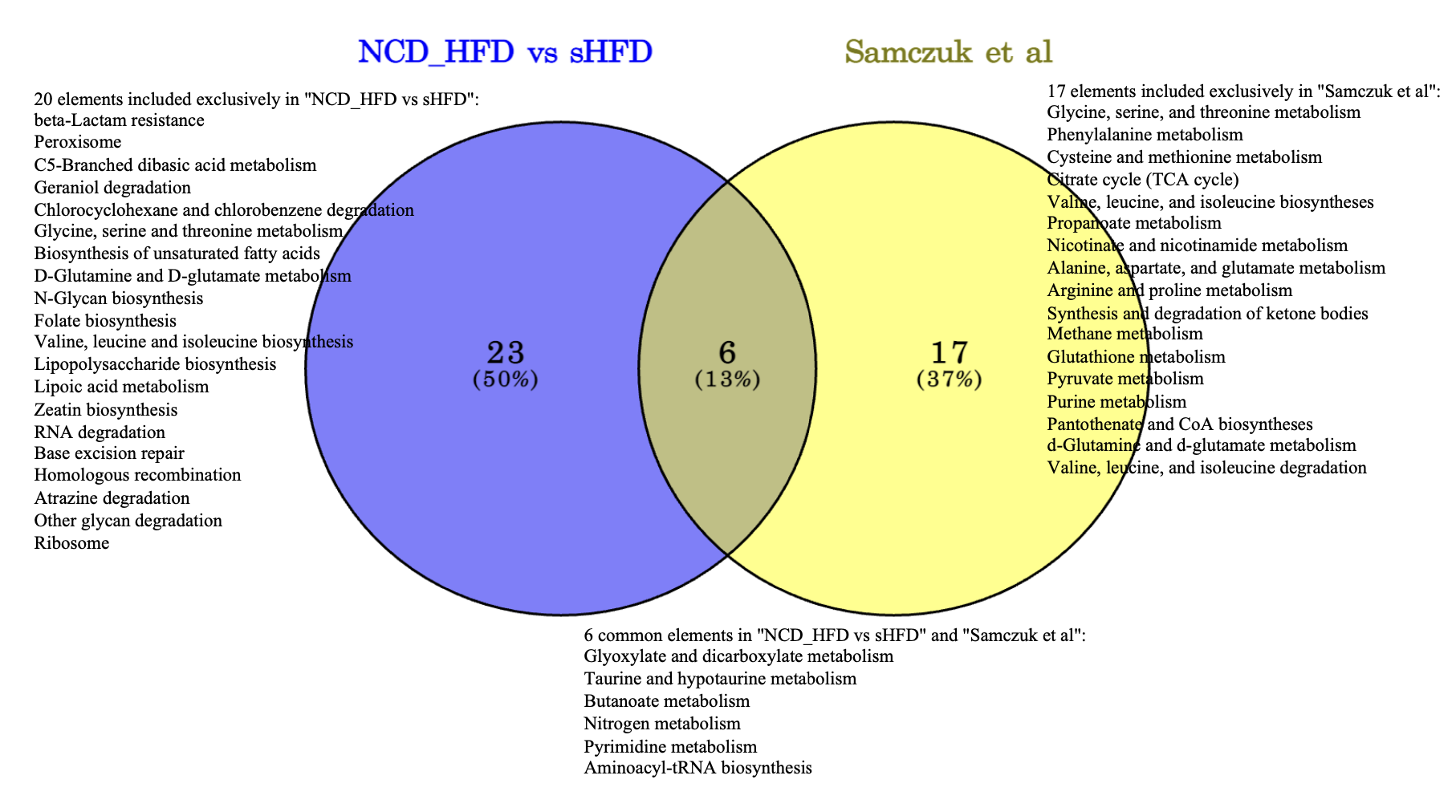


**Supplementary Figure S10.** The comparison of our results with those of Samczuk et al on metabolic pathways after bariatric surgery (p < 0.05)


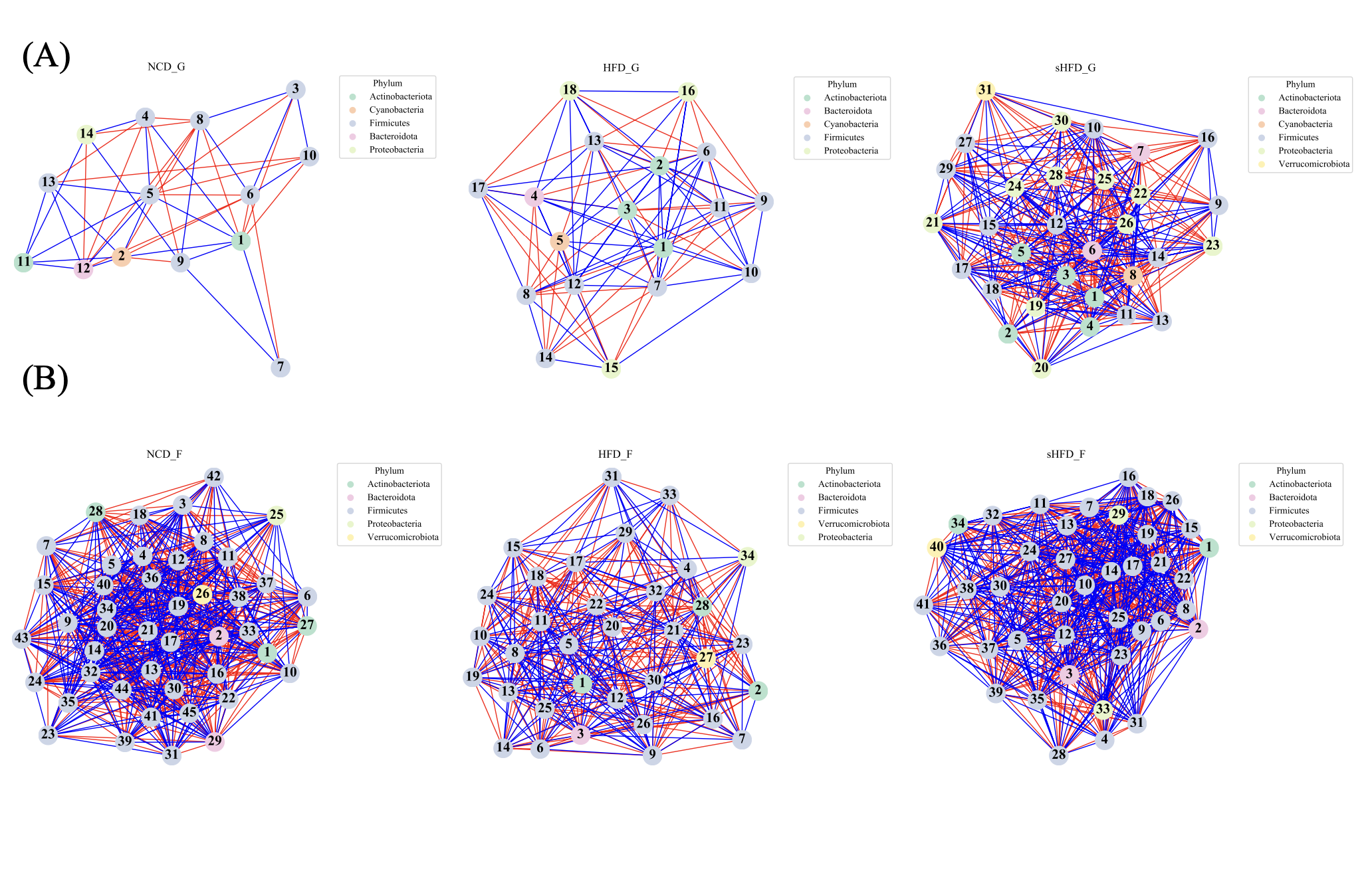


**Supplementary Figure S11.** The co-occurrence network of whole microbiota genera from NCD, HFD and sHFD group in gastric and fecal

Co-occurrence plots are shown for the gastric (A)- and fecal(B)-derived microbiota of rats fed the normal chow diet (NCD), rats fed a high-fat diet (HFD), and HFD-induced rats that underwent sleeve gastrectomy.
